# Malignant transformation in patients with monoclonal gammopathy of undetermined significance treated with teriparatide for osteoporosis: a bicenter retrospective study and analysis of the French national pharmacovigilance database

**DOI:** 10.1007/s11657-026-01693-x

**Published:** 2026-03-29

**Authors:** R. Cassez, B. Cortet, B. Bouvard, D. Theis, C. Potey, S. Manier, J. Paccou, C. Philippoteaux

**Affiliations:** 1 Rheumatology Department, Lille University Hospital, 59000 Lille, France; 2 Rheumatology Department, Angers University Hospital, 49000 Angers, France; 3 Department of Medical Information, Lille University Hospital, 59000 Lille, France; 4 Department of Pharmacovigilance, Lille University Hospital, 59000 Lille, France; 5 Department of Hematology, Lille University Hospital, 59000 Lille, France

**Keywords:** Osteoporosis, Teriparatide, MGUS, Multiple myeloma, Malignancy

## Abstract

***Summary*:**

Malignant transformation in patients with monoclonal gammopathy of undetermined significance treated with teriparatide was evaluated. Among 29 patients, only two progressed to hematologic malignancy (one multiple myeloma, one Waldenström’s macroglobulinemia). These findings are reassuring and suggest that TPT may remain an option for selected patients with severe osteoporosis and MGUS.

**Purpose:**

Osteoporosis is a highly prevalent bone disease with limited therapeutic options, particularly in multimorbid patients. Monoclonal gammopathy of undetermined significance (MGUS), a frequent finding in osteoporosis and a recognized fracture risk factor, often leads to contraindication of teriparatide (TPT), further restricting treatment choices. This study aimed to assess hematologic malignant transformation in osteoporotic patients with MGUS treated with TPT.

**Methods:**

A retrospective bicenter observational study was conducted at Lille and Angers University Hospitals (2016–2022). Patients with osteoporosis and confirmed MGUS at TPT initiation were included. Demographic, hematologic, bone, and treatment parameters were collected. In parallel, hematologic malignancies reported with TPT were reviewed from the French national pharmacovigilance database (PVDB) over the same period.

**Results:**

Twenty-nine patients (69% women; mean age 72.6 ± 12.6 years) were included. Most had major comorbidities (89.7% with Charlson Comorbidity Index ≥ 3). MGUS was IgG-type in 55.2% and IgM-type in 41.4%. The mean follow-up from TPT initiation was 60.2 ± 30.7 months (median 54.0; 95% CI 49.0–71.4). Two patients developed hematologic malignancy: one multiple myeloma (MM) 7 years after TPT and one Waldenström’s macroglobulinemia 21 months after initiation, both with intermediate-risk MGUS. TPT was well tolerated with premature discontinuation in 17.2% of cases. Of the two hematologic malignancies identified in the PVDB, only one (MM) occurred in a patient with pre-existing MGUS.

**Conclusion:**

Malignant progression was rare, providing reassuring evidence regarding hematologic safety. TPT may be considered in selected patients with severe osteoporosis and MGUS, provided careful hematologic monitoring.

**Graphical abstract:**

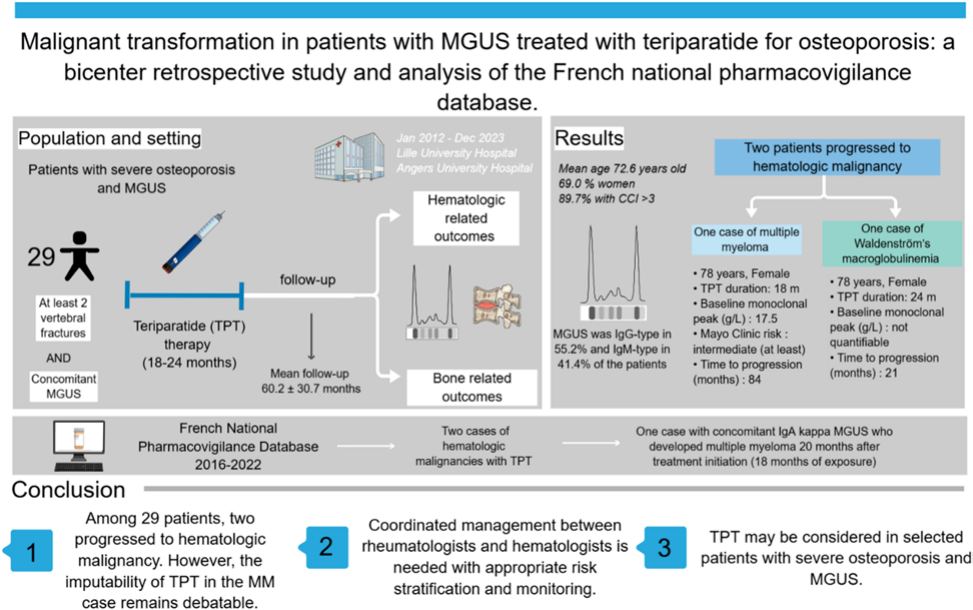

**Supplementary Information:**

The online version contains supplementary material available at 10.1007/s11657-026-01693-x.

## Introduction

Osteoporosis is a systemic skeletal disease, characterized by reduced bone mass and altered bone tissue microarchitecture, leading to an increased fragility and fracture risk [1]. Its high prevalence worldwide makes it a major public health issue, due to its impact on morbidity, mortality, and quality of life [2–4]. The emergence of innovative treatments has improved fracture prevention and restored bone quality [5–7]. Among existing treatments, teriparatide (TPT), a recombinant fragment of human parathyroid hormone (PTH), plays a central role in the management of severe forms of the disease [8–10]. Monoclonal gammapathy of unknown significance (MGUS) is a benign clonal abnormality of bone marrow plasma cells, with a high prevalence increasing with age (5% of people over 70 and 9% of people over 85) [11], with no organ involvement [12], with an estimated risk of progression to multiple myeloma (MM) of 1% per year [13]. Beyond its frequent coexistence with osteoporosis in older patients, MGUS itself has been associated with increased skeletal fragility in large population-based with hazard ratios ranging from 1.29 (95% CI 1.21–1.37) for overall fractures [14] to a relative risk of 2.5 (95% CI 1.53–4.06) for vertebral fractures (VF) [15]. In addition, high-resolution peripheral quantitative computed tomography (HR-pQCT) studies have shown altered bone microarchitecture, particularly affecting trabecular bone, and reduced bone strength in MGUS patients [16]. Therefore, TPT may be particularly relevant in this setting because of its anabolic effects on osteoblast function and trabecular bone [17].

Mechanistically, TPT stimulates osteoblast activity through activation of the parathyroid hormone 1 receptor (PTH1R) and the Wnt/β-catenin pathway, promoting bone formation and a positive bone mass balance [18–20]. Nevertheless, this mechanism theoretically carries a risk of solid tumor promotion by enhancing malignant cell proliferation, angiogenesis, and bone resorption via cytokines such as interleukin-6 (IL-6), RANKL, and tumor necrosis factor-α (TNF-α) [21, 22]. Accordingly, TPT is contraindicated in patients with a history of neoplasia, particularly bone tumors or cancers with bone metastases [23, 24].

The hypothesis of a deleterious effect of TPT on MGUS progression stems from plausible biological arguments [25, 26], including alterations of the bone microenvironment, vasodilatory effects of PTH, and preclinical oncogenic data [27, 28]. However, these concerns primarily arose from a theoretical risk of osteosarcoma development in solid tumors or bone metastases rather than hematologic malignancies [29]. Importantly, no such signal has been confirmed in humans when TPT is used within current recommendations [27, 30].

In this context, the use of TPT in patients with MGUS remains debated. Available clinical data are scarce, and no causal link between TPT and progression of MGUS or plasma cell disorders has been demonstrated. Isolated case reports call for caution but are inconclusive [31, 32]. In France, practices remain heterogeneous: some centers use TPT in selected MGUS patients, whereas others adopt a more conservative approach. Hematologists often play a consultative role, but no formal contraindication has been issued.

## Purpose

Given the lack of available data, this study aims to assess MGUS progression in a cohort of osteoporosis patients treated with TPT, focusing on incidental malignant hematologic cases. Secondary endpoints included evaluating bone outcomes (BMD, fractures), treatment tolerability, and MGUS monitoring to optimize TPT use and ensure safety in targeted populations. We completed our study with the analysis for hematologic malignancies with TPT reported in the French nationwide pharmacovigilance database.

## Methods

### Study design and population

The STROBE guideline was followed [33]. A retrospective observational bi-centered (Lille University Hospital, Angers University Hospital, France) study was conducted from January 1 st, 2016, to December 31, 2022. Patients aged ≥ 18 years with confirmed osteoporosis and a concomitant diagnosis of MGUS who received TPT and were followed in the Rheumatology department were included.

MGUS was defined according to International Myeloma Working Group (IMWG) criteria [34]: (i) serum monoclonal protein < 30 g/L, (ii) < 10% bone marrow plasma cells, and (iii) absence of myeloma-defining events (hypercalcemia, anemia, renal failure, or lytic bone lesions). Osteoporosis was severe in all cases, with ≥ 2 VFs, fulfilling French criteria for TPT prescription. TPT was initiated either at Lille University Hospital, Angers University Hospital (one patient), or by external rheumatologists, with all patients subsequently followed in the rheumatology departments of Lille or Angers.

### Ethical consideration

As a retrospective study, an Ethics Committee and Institutional Review Board and informed consent from patients were not required, according to French law (JORF number 160 13th July 2018). The study was performed in compliance with MR-004, received permission from Lille University Hospital, and was declared to the French Data Protection Authority (reference DEC24-289).

### Study assessment and data collection

Data were retrieved from the Lille University Hospital health data warehouse (INCLUDE) and patients’ electronic medical records. The Medical Information Department performed an initial extraction (2016–2022) using keyword searches in databases and clinical notes.

Subsequent manual chart reviews extended this search period, allowing the identification of patients whose TPT therapy had been initiated as early as July 2012**.** Dates of TPT initiation in the final cohort therefore ranged from July 2012 to January 2023, and follow-up was extended until February 2025 to ensure adequate observation of treatment outcomes and potential malignant transformations. Keywords for treatment included *teriparatide*, *Forsteo*, *Movymia*, *Terrosa*, *Livogiva*, and *Sondelbay*; for MGUS, the terms were *dysglobulinemia*, *monoclonal gammopathy*, and *MGUS*. Each hit was assessed within a 200-character context window to ensure relevance. Records were then manually reviewed to confirm concomitant TPT therapy and MGUS, with strict application of inclusion criteria. The initial query identified 101 patients, but after exclusions (e.g., contraindication to TPT because of MGUS), only those with confirmed MGUS receiving TPT were retained in the final cohort.

### Definitions

#### Osteoporosis and fragility fracture

Densitometric osteoporosis was defined according to the official positions of the International Society for Clinical Densitometry (ISCD) and the World Health Organization (WHO) classification [35, 36]. In postmenopausal women and men aged ≥ 50 years, osteoporosis corresponded to a *T*-score ≤  − 2.5 SD at the lumbar spine, total hip, or femoral neck. In premenopausal women and men < 50 years, *Z*-scores were used, with values ≤  − 2.0 SD classified as below the expected range for age. A fragility fracture was defined as a low-trauma fracture, typically resulting from a fall from standing height or less. Eligible fracture types included VF, hip fractures, severe non-hip/non-vertebral fractures (proximal humerus, pelvis, distal femur, proximal tibia, or ≥ 3 rib fractures), and non-severe fractures (e.g., wrist, elbow, ankle). Regarding bone mineral density (BMD) measurements, during the study period, two Hologic© scanners were available at Lille University Hospital (HOLOGIC Discovery A S/N 81360 and HOLOGIC Horizon W S/N 300869 M).

#### Teriparatide use

Data on TPT use included initiation and discontinuation dates, total cumulative treatment duration (months), and any premature discontinuation with its underlying reason (when available). The occurrence of new fragility fractures during and after TPT therapy was recorded, specifying the anatomical site and date of each event. Post-treatment BMD measurements were collected, with particular attention to assessments performed approximately 18 months after initiation. Finally, subsequent osteoporosis therapies prescribed after TPT cessation were documented.

#### MGUS diagnosis

For MGUS, the origin of the diagnosis was noted, whether established by a hematologist or identified by a rheumatologist during the systematic evaluation of fragility-related osteoporosis, which included serum protein electrophoresis (SPE). Variables collected comprised MGUS subtype, monoclonal spike level, serum free light chain (FLC) ratio, bone marrow biopsy results when available, and disease severity according to the Mayo Clinic risk classification [34, 37]. Hematologic follow-up was documented through changes in the monoclonal spike and the availability of longitudinal monitoring. Progression to MM or other hematologic malignancies, such as Waldenström’s macroglobulinemia, was recorded when applicable.

### Patient demographics and comorbidities

Clinical data were manually extracted through a comprehensive review of each patient’s electronic medical records. Collected variables included age at TPT initiation, sex, body mass index (BMI), and major comorbidities: hypertension, diabetes mellitus, stroke or transient ischemic attack (TIA), myocardial infarction, obstructive sleep apnea (OSA), chronic kidney disease (eGFR < 60 mL/min), chronic obstructive pulmonary disease (COPD) or respiratory insufficiency, solid organ malignancy, dyslipidemia, atrial fibrillation, heart failure, peripheral arterial disease, Parkinson’s disease, dementia, hematologic malignancies (leukemia or lymphoma), and cirrhosis. These data allowed calculation of the Charlson Comorbidity Index (CCI) [38]. Osteoporosis-specific risk factors were also recorded, including current or past smoking, excessive alcohol consumption (≥ 3 alcohol units/day for men or ≥ 2 units/day for women), family history of fragility fractures—particularly hip fractures in first-degree relatives—personal history of fragility fractures, and age at menopause in women (early menopause defined as permanent amenorrhea before age 40). Information on medications associated with increased osteoporosis risk was collected, including corticosteroids, proton pump inhibitors (PPIs), selective serotonin reuptake inhibitors (SSRIs), anticonvulsants, and hormonal replacement therapy. Vitamin D status was documented when available, with insufficiency defined as serum 25-hydroxyvitamin D < 30 ng/mL. Some factors, such as history of falls, were not systematically recorded and therefore were not included in the analysis.

### Outcome measure

The primary endpoint was the incidence of malignant transformation in a cohort of osteoporotic patients with MGUS treated with TPT. The diagnosis of MM followed IMWG criteria [12]: identification of a monoclonal immunoglobulin in serum and/or urine and/or bone marrow plasma cell infiltration ≥ 10%, with at least one CRAB criterion and/or a biomarker of malignancy (defining symptomatic MM), or, in the absence of symptoms, with a monoclonal immunoglobulin concentration > 30 g/L and/or bone marrow plasmacytosis ≥ 10% (defining smoldering MM).

Secondary endpoints included bone outcomes, assessed by the occurrence of new fragility fractures and BMD changes, as well as adverse effects related to TPT.

### Pharmacovigilance analysis

To complement our cohort study, we reviewed data from the French National Pharmacovigilance Database (2016–2022). All cases in which TPT was reported as a suspected drug and associated with cancer occurrence were extracted. Clinical narratives were analyzed to identify patient characteristics, type of malignancy (solid or hematologic), treatment duration, and latency between TPT initiation and cancer diagnosis. Duplicate reports were excluded after cross-checking anonymized identifiers.

### Statistical analyses

Descriptive statistics were used to summarize the study population. Continuous variables were presented as means with standard deviations (SD) or as medians with interquartile ranges (IQR), depending on their distribution. Normality was assessed using histograms and the Shapiro–Wilk test. Paired Student’s *t*-tests were applied for normally distributed paired data, and the Wilcoxon signed-rank test was used otherwise. Categorical variables were reported as frequencies and percentages, with comparisons performed using Fisher’s exact test. Longitudinal changes in bone densitometry and biological parameters were analyzed using paired tests according to normality assumptions. A binary logistic regression model was employed to assess the association between treatment duration and fracture occurrence during follow-up. All statistical tests were two-sided, with *p* < 0.05 considered statistically significant. Analyses were performed on complete cases using IBM SPSS Statistics, version 29.0.0.0.

## Results

### Study population

#### Demographic data and comorbidities

**Fig. 1:**
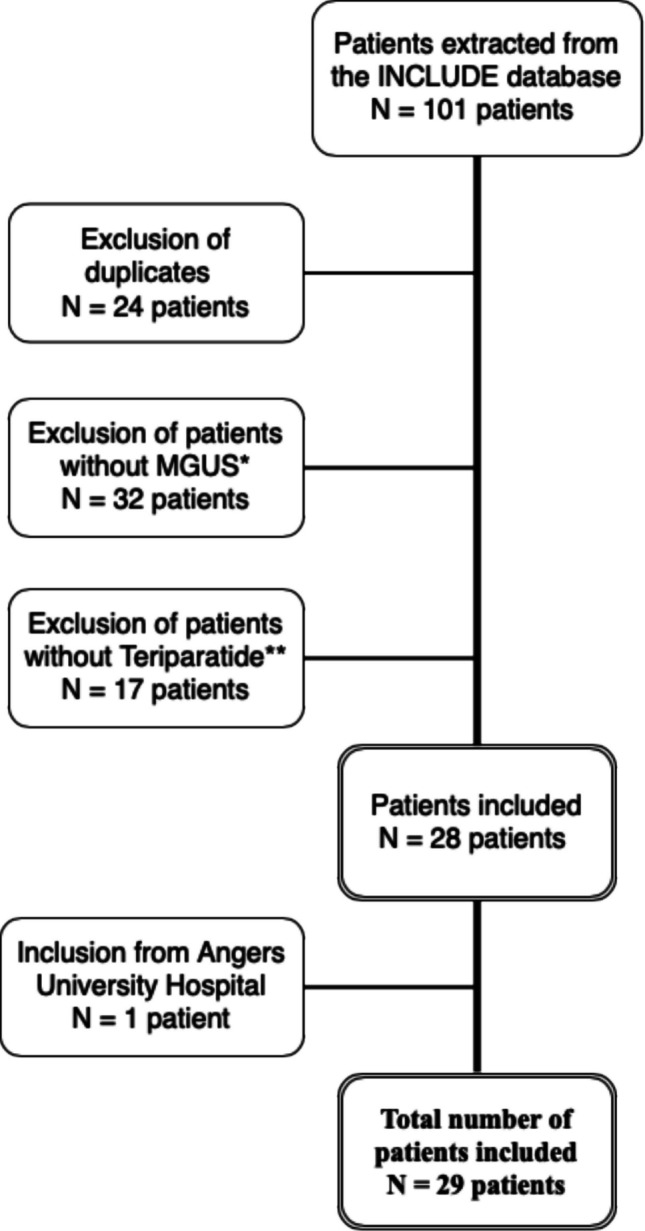
Study flowchart. *Among patients: MGUS diagnosis not confirmed: *N* = 25 patients; MGUS discovered after TPT initiation: *N* = 6 patients; diagnosis of multiple myeloma at time of introduction: *N* = 1. **Among patients: TPT not indicated according to recommendations: *N* = 6 patients (5 because of radiotherapy or cancer); TPT recommended but finally not introduced due to MGUS: *N* = 5 patients; TPT indicated but patients refused: *N* = 4 patients; Allergic reaction to introduction: *N* = 1 patient; misinformation: *N* = 1 patient

A total of 29 patients were included (Fig. 1), of whom 20 (69.0%) were women. The mean age was 72.6 ± 12.6 years, and the mean BMI was 26.6 ± 6.2 kg/m^2^ (Table 1). The most frequent comorbidities were hypertension (*n* = 16, 55.2%), dyslipidemia (*n* = 12, 41.4%), COPD or respiratory insufficiency (*n* = 8, 27.6%), diabetes mellitus (*n* = 5, 17.2%), and heart failure (*n* = 4, 13.8%). Most patients (*n* = 26, 89.7%) had a CCI > 2, with a median of 5 (range, 1–11) (Table 1). Regarding lifestyle factors, 10 patients (34.5%) were current or former smokers and 6 (20.7%) reported excessive alcohol consumption.

**Table 1:** Baseline characteristics of study population

Parameters	Available data	*N* (%)
**Sex** Men Women	29	9 (31) 20 (69)
**Age (years) mean (SD)**	29	72.6 (12.6)
**BMI (kg/m**^**2**^**) mean (SD)**	29	26.6 (6.2)
**Charlson comorbidity index** 1–2 > 2 **Comorbidities**	29 29	3 (10.3) 26 (89.7)
HBP		16 (55.2)
Diabetes mellitus		5 (17.2)
Stroke or TIA		4 (13.8)
Myocardial infarction		3 (10.3)
OSA		5 (17.2)
Chronic renal failure		0
COPD or chronic respiratory failure		8 (27.6)
Solid neoplasia		2 (6.9)
Dyslipidemia		12 (41.4)
Heart failure		4 (13.8)
PAOD		2 (6.9)
Dementia		3 (10.3)
Cirrhosis		4 (13.8)
**Smoking status** Non-smoker Current smoker Former smoker	29	19 (65.5) 3 (10.3) 7 (24.1)
**Alcohol status** Non-alcohol Active or weaned chronic alcoholism	29	23 (79.31) 6 (20.7)
IRD/connective tissue disorder *	29	8 (27.6)
**Malabsorptive pathology or bariatric surgery**	29	4 (13.8)
Endocrinopathy **	29	2 (6.9)
**Fracture history**	29	
2 VFs > 2 VFs Hip fractures Proximal humerus Wrist		5 (17.2) 24 (82.8) 4 (13.8) 3 (10.3) 3 (10.3)
**Other osteoporosis risk factors**		
Vitamin D deficiency (< 30 ng/ml) Family history of hip fracture Early menopause	26 29 29	13 (44.8) 2 (6.9) 2 (6.9)
History of osteoporosis-inducing treatment	29	22 (75.9)

Values are expressed as number (%) unless otherwise stated. Abbreviations: *HBP*, high blood pressure; *TIA*, transient ischemic attack; *OSA*, obstructive sleep apnea; *COPD*, chronic obstructive pulmonary disease; *PAOD*, peripheral arterial occlusive disease; *VF*, vertebral fracture

^*^5 cases of chronic inflammatory rheumatism, 3 cases of connective tissue disorder. **1 case of hyperthyroidism and 1 case of hyperparathyroidism. ***9 patients receiving corticosteroids, 15 receiving proton pumps inhibitors, 2 receiving anticonvulsants, 5 selective serotonin reuptake inhibitors, 3 receiving hormone therapy

#### MGUS at baseline

Of the 29 patients, 28 (96.6%) had a monoclonal peak identified prior to TPT initiation; in one patient, the peak was detected immediately after initiation and considered baseline. A quantifiable peak on SPE was present in 15 patients (51.7%), with a mean baseline value of 6.78 ± 6.76 g/L (Table 2). MGUS was first identified during the pre-treatment work-up by a rheumatologist in 14 patients (48.3%), by a hematologist in 9 (31.0%), and by other specialists in 6 (20.7%). Following detection of the monoclonal peak, 11 patients (37.9%) underwent hematologic follow-up, defined as ≥ 1 consultation with a hematology specialist.

**Table 2:** Main characteristics of the MGUS at teriparatide introduction

Parameters	Available data (*N*)	Before TPT
Median monoclonal peak (g/L) [Q1–Q3]	15	3.40 [1.90–4.90]
Mean monoclonal peak (g/L) (SD)	15	6.78 ± 6.76
MGUS type	29	
IgG kappa		8 (27.6)
IgG lambda		8 (27.6)
IgM		12 (41.4)
IgA		1 (3.4)
Abnormal FLC ratio	17	7 (41.2)
Mayo Clinic MGUS risk	17	
Low risk (0 factor)		7 (41.2)
Intermediate risk (1 factor)		8 (47.1)
High risk (2 factors)		2 (11.8)
Bone marrow biopsy performed, n (%)	6	6 (25.0)
Specialist who made MGUS diagnosis	29	
Hematologist		9 (31.0)
Rheumatologist		14 (48.3)
Other		6 (20.7)

Values are expressed as number (%) unless otherwise stated

Abbreviations: *FLC*, free light chains; *IgA*, immunoglobulin A; *IgG*, immunoglobulin G; *IgM*, immunoglobulin M; *MGUS*, monoclonal gammopathy of undetermined significance; *Q1–Q3*, first and third Quartiles; *SD*, standard deviation; *TPT*, teriparatide

Regarding MGUS subtypes, 16 cases (55.2%) were IgG (8 IgG kappa (27.6%) and 8 IgG lambda (27.6%)), 12 (41.4%) were IgM, and 1 (3.4%) was IgA. Among the 17 patients who underwent serum FLC analysis, 7 (41.2%) had an abnormal FLC ratio. Based on the Mayo Clinic risk stratification model, 7 patients (41.2%) were classified as low risk (0 factor), 8 (47.1%) as intermediate risk with 1 factor, and 2 (11.8%) as intermediate risk with 2 factors; none had all 3 factors associated with high risk. A bone marrow biopsy was performed in six patients (20.7%), all showing plasma cell infiltration below 10%, with no cytologic atypia or features suggestive of clonal progression, thus confirming the diagnosis of MGUS.

#### Osteoporosis at baseline

All 29 patients had a history of at least two VFs, including 24 (82.8%) with more than two. Additional fracture sites included the proximal femur in 4 patients (13.8%), the proximal humerus in 3 (10.3%), and the wrist in 3 (10.3%). Osteoporosis risk factors included a first-degree family history of proximal femur fracture in 2 patients (6.9%) and early menopause in 2 (6.9%). Vitamin D insufficiency was documented in 13 patients (44.8%), and 2 (6.9%) had endocrinopathies (one hyperparathyroidism, one hyperthyroidism).

Medications associated with increased osteoporosis risk were frequent: 9 patients (31.0%) had long-term corticosteroid therapy, 15 (51.7%) used PPIs, 5 (17.2%) were on SSRIs, 3 (10.3%) had received hormonal replacement therapy, and 2 (6.9%) were treated with anticonvulsants (levetiracetam). For 12 patients (41.4%), TPT was not the first-line osteoporosis treatment. Among them, 11 received TPT as second-line therapy (10 (91.7%) after bisphosphonates, 1 (8.3%) after denosumab), and 1 patient (3.4%) as third-line after sequential bisphosphonates and denosumab.

Baseline BMD assessment was available for most patients, though completeness varied by site. At baseline, lumbar spine *T*-score was available for 19 patients (65.5%), femoral neck *T*-score for 19 patients (65.5%), and total hip *T*-score for 16 patients (55.2%), with mean values of −1.4, −2.0, and −1.6 SD, respectively. BMD data were missing for several patients, for multiple reasons: in some cases, the medical records mentioned that DXA had been performed but without reporting numerical results; in others, the examination was planned but not completed, or performed externally without available digital data. In several instances, spine BMD was deemed uninterpretable by the prescriber due to multiple vertebral fractures or advanced degenerative changes, explaining the higher proportion of missing lumbar measurements.

#### Focus on the profile of patients recused from teriparatide due to MGUS

Five patients with severe osteoporotic fractures fulfilled the theoretical criteria for TPT but were excluded by the rheumatologist in charge, specifically because of MGUS detected during baseline screening (Fig. 1). They were predominantly women (4/5), older (64–89 years), and highly comorbid (Supplementary Table 1). All five patients had multiple VF (at least two), sometimes associated with peripheral fractures such as the wrist, sacrum, or metatarsals. BMD values ranged from moderately reduced (spine *T*-score −0.4) to severely osteoporotic levels (spine *T*-score −4.8, femoral neck −2.1. MGUS subtypes were mainly IgG kappa (3 cases), with one IgM kappa and one IgA lambda. In all cases, MGUS was the explicit reason for TPT contraindication, and patients were redirected to antiresorptive (zoledronate, denosumab, or bisphosphonates). Hematologic follow-up was inconsistent, being absent in several cases or limited to general practitioner monitoring. Based on the available data, no patient showed clinical or biological progression to multiple myeloma or another hematologic malignancy during follow-up.

### Malignant evolution

The mean follow-up duration from TPT initiation to the end of data collection (February 2025) was 60.2 ± 30.7 months (median 54.0, 95% CI 49.0–71.4). At the end of TPT treatment, monoclonal peak values were available for 19 patients (65.6%), including 11 (57.9%) with a quantifiable peak on SPE. The mean post-treatment peak was 9.61 ± 8.82 g/L. Compared with baseline, this represented a significant mean increase of 2.83 g/L (95% CI, 0.68–4.99; *p* = 0.021). Although outliers were present, the overall distribution showed a higher median and an upward shift.

During the follow-up period, 2 patients progressed to hematologic malignancy: one to MM and one to Waldenström’s macroglobulinemia, defined by clonal lymphoplasmacytic bone marrow infiltration, non-systematic clonal lymphocytosis, and secretion of an IgM-type monoclonal immunoglobulin (Table 3). No malignant transformation was observed in the remaining 27 patients, regardless of changes in monoclonal peak levels.

**Table 3:** Main characteristics of patients with malignant transformation

Characteristics	Case 1: multiple myeloma	Case 2: Waldenström’s macroglobulinemia
Age at TPT initiation	78 years	78 years
Sex	Female	Female
Charlson Comorbidity Index (CCI)	4	5
Relevant medical history	Systemic sarcoidosis, multiple VFs	Obesity, hypertension, OSAS, SLE, VF + hip fracture
Alcohol/tobacco use	None	None
Type of MGUS	IgG lambda	IgM
Baseline monoclonal peak (g/L)	17.5	Not quantifiable
Light chain ratio	Not available	Normal
Mayo Clinic risk category	At least intermediate risk	Intermediate risk (1 factor)
Bone marrow biopsy at MGUS diagnosis	Not performed	Not performed
TPT duration	18 months	24 months
Subsequent anti-osteoporotic treatment	Risedronate	Zoledronic acid
Fractures during follow-up	None	None
BMD (*T*-score)	LS −1.5, FN −3.1, TH −2.3	Not available
Signs of progression	L1 focal lesion, Bence Jones proteinuria	General decline, non-regenerative anemia
Final monoclonal peak (g/L)	24.7	5.7
Bone marrow infiltration at progression	35%	20%
Time to progression (months)	84	21
Hematologic malignancy	Multiple myeloma	Waldenström’s macroglobulinemia

Abbreviations: *BMD*, bone mineral density; *CCI*, Charlson comorbidity index; *FN*, femoral neck; *LS*, lumbar spine; *MGUS*, monoclonal gammapathy of unknown signification; *OSAS*, obstructive sleep apnea syndrome; *SLE*, systemic lupus erythematous; *TH*, total hip; *TPT*, teriparatide; *VF*, vertebral fracture

#### Case of progression to multiple myeloma

This case involved a 78-year-old woman (CCI 4) with systemic sarcoidosis and osteoporosis complicated by multiple VFs. Baseline BMD was unavailable because it was not referenced in the medical file. An IgG lambda MGUS with a monoclonal peak of 17.5 g/L had been identified prior to treatment. The patient had been followed in hematology since 2010, with several annual consultations confirming stable MGUS based on consistent laboratory results. The serum FLC ratio was not reported, precluding full Mayo Clinic risk stratification, although the elevated peak indicated at least an intermediate-risk profile. No myelogram was performed at diagnosis.

TPT was initiated in July 2012 and continued for 18 months, followed by denosumab. No new fractures occurred during follow-up, but post-treatment *T*-scores (after 18 months) confirmed persistent low BMD values (−1.5 lumbar spine, −3.1 femoral neck, −2.3 total hip). In 2014, 2 years after TPT initiation, Bence Jones proteinuria (PBJ) appeared “at trace levels,” prompting continued monitoring without treatment. Follow-up in 2015 confirmed persistence of the MGUS with therapeutic abstention, and the PBJ was negative again in 2016. The patient was subsequently lost to follow-up for 3 years. In 2019, she re-presented with anemia (Hb 10.6 g/dL, non-regenerative), a progressive rise in the monoclonal peak (24.7 g/L), and reappearance of Bence Jones proteinuria (0.54 g/L). A myelogram demonstrated 35% plasma cell infiltration, confirming IgG lambda MM meeting CRAB criteria (anemia and lytic bone lesions on MRI). The interval between TPT initiation and MM diagnosis was 84 months (7 years).

#### Case of progression to Waldenström’s macroglobulinemia

The second case concerned a 78-year-old woman with obesity, hypertension, OSA, and systemic lupus erythematosus (SLE). Osteoporosis was complicated by two FVs and a femoral neck fracture. BMD values were unavailable in the medical files. Risk factors included prolonged corticosteroid use (SLE-related) and chronic PPI use. An IgM-type MGUS had been identified prior to treatment, characterized by a non-quantifiable monoclonal peak, normal light chain ratio, and an intermediate-risk Mayo Clinic profile. TPT was initiated in July 2020 and continued for 24 months, followed by a single infusion of zoledronic acid. No new fractures occurred during therapy. In February 2022, the patient progressed to Waldenström’s macroglobulinemia, presenting with clinical deterioration and non-regenerative anemia. The monoclonal peak had increased to 5.7 g/L, and bone marrow examination showed 20% infiltration by lymphoplasmacytic cells.

### Fracture incidence and BMD variation

#### BMD changes

Changes were analyzed only when baseline and follow-up measurements were performed on the same DXA machine. After TPT treatment, the mean lumbar spine *T*-score was −0.78 ± 1.42 SD, showing a significant mean gain of + 0.66 SD, corresponding to a + 6.7% BMD increase (95% CI [0.24–1.09]; *p* = 0.012). No statistically significant variations were observed at other skeletal sites, with mean femoral neck *T*-score −2.0 ± 0.9 SD (BMD −1.8%; 95% CI [−6.2%; + 2.6%]) and mean total hip *T*-score −1.8 ± 0.9 SD (BMD −2.0%; 95% CI [−7.1%; + 3.1%]).

#### Fracture incidence

Among the 29 patients treated with TPT, 4 (13.8%) experienced at least one new fracture during or after TPT therapy, all confirmed by imaging: one hip fracture, one case of multiple VFs, one isolated VF, and one wrist fracture followed by a subsequent hip fracture.

### Therapeutic sequence and side effects

Treatment was prematurely discontinued in 5 of 29 patients (17.2%). TPT was administered for exactly 18 months in 18 patients (62.1%) and for > 18 months in 4 patients (13.8%); no patient exceeded the recommended 24-month limit. The mean treatment duration was 16.7 months. Causes of discontinuation included premature death (*n* = 3), musculoskeletal pain (*n* = 1), and gastrointestinal symptoms associated with headache (*n* = 1).

Three patients were lost to follow-up after TPT treatment. Among the remaining 26, 21 (80.8%) transitioned to antiresorptive therapy: 12 received zoledronic acid, 6 denosumab, and 3 oral bisphosphonates (risedronate or alendronate), consistent with current post-anabolic management recommendations (Supplementary Fig. 1).

### Results from the French national pharmacovigilance database

Review of the French National Pharmacovigilance Database identified eight cases of cancer associated with TPT. Six were solid tumors (breast, kidney, endometrium, colon, chondrosarcoma, and basal cell carcinoma), diagnosed between 9 and 17 months after treatment initiation. Two cases involved hematologic malignancies. The first concerned a 71-year-old woman with a history of IgA kappa MGUS who developed MM 20 months after treatment initiation (18 months of exposure). She presented with normocytic, non-regenerative anemia (< 10 g/dL), a monoclonal spike of 6.3 g/L, vertebral lesions on CT, and bone marrow infiltration of 17% plasma cells, leading to the diagnosis; therapeutic abstention with close monitoring was chosen. The second case was a 70-year-old woman with giant cell arteritis and refractory anemia with excess blasts (RAEB), treated with TPT for glucocorticoid-induced osteoporosis; after 2 months of therapy, RAEB progressed to acute myeloid leukemia.

## Discussion and conclusion

### Main results

In our cohort of 29 MGUS patients treated with TPT (69% women, mean age 72.6 years), the mean follow-up duration was 60.2 ± 30.7 months (median 54.0; 95% CI 49.0–71.4). MGUS was mainly IgG (55.2%) or IgM (41.4%). Two patients progressed to hematologic malignancy, one MM and one Waldenström’s macroglobulinemia, both with intermediate-risk MGUS at baseline. TPT was generally well tolerated, with premature discontinuation in 17.2% of cases, and lumbar spine BMD improved significantly (+ 0.66, *p* = 0.012). Among the two cases of malignant hematologic disorders associated with TPT identified in the French national pharmacovigilance database, only one occurred in a patient with pre-existing MGUS (MM).

### Discussion from literature

*Regarding demographic data in our study*, the proportion of women (69%) aligns with the typical postmenopausal demographic of osteoporosis. The mean age was 72.6 ± 12.6 years, consistent with the age group at highest fracture risk and comparable to MGUS cohorts, such as that of Kyle et al., which shows that the prevalence of MGUS increases with age, suggesting that the mean age of affected patients is around 70 (21,463 individuals from Minnesota in the USA underwent SPE, identifying 694 cases of MGUS) [11]. The median CCI in our cohort was 5, with 89.7% of patients associated with severe comorbidities (CCI > 2), highlighting a multimorbid population. The patient’s profile and comorbidities were consistent with those reported in large MGUS cohorts, such as that of Epstein et al. [39].

*Regarding the characteristics of the MGUS*, most patients had an IgG isotype, consistent with epidemiological data showing that IgG MGUS accounts for 60–70% of cases [13]. The mean M-protein level was relatively low (6.78 g/L; median 4.7 g/L), and 41% of patients with available data had an abnormal FLC ratio, in line with Rajkumar et al. where approximately one-third of MGUS patients showed altered FLC values [37]. Missing evaluations (FLC, bone marrow) limited risk stratification, but 17 patients were classifiable, mostly as low or intermediate risk. Nearly half of MGUS cases were detected during rheumatology-led osteoporosis assessments, highlighting their value for uncovering latent hematologic disorders. This is consistent with Bouvard et al., who reported concomitant MGUS and osteoporosis in about one-quarter of cases [40]. However, only 38% of patients received hematology follow-up, underscoring heterogeneous practices and possible underestimation of progression risk.

*When considering the hematologic evolution during follow-up*, a significant increase in the monoclonal peak was observed (+ 2.83 g/L after treatment). Two patients progressed to hematologic malignancy: one to symptomatic MM diagnosed 7 years after a 24-month TPT course and one to Waldenström’s macroglobulinemia 21 months after initiation. In the first case, transient trace Bence Jones proteinuria appeared 2 years after TPT and later resolved, with transformation documented only in 2019, consistent with the natural course of MGUS rather than treatment exposure. In large MGUS cohorts, the annual progression risk is estimated at 0.5–1% [13, 37], and no progression was observed in the remaining 93% of our patients, which is overall reassuring. To date, among the four published cases of hematologic malignancy temporally associated with TPT [31, 32, 41, 42] one explicitly involved a pre-existing MGUS, to which we add the two patients from our series and one PBDV case, yielding four cases of malignant transformation from known MGUS (Table 4). The hematologic outcomes included three MM and one Waldenström’s macroglobulinemia. Among the four cases with explicitly documented pre-existing MGUS, the mean age at malignant progression was 71.5 ± 9.0 years (95% CI 57.2–85.8). The mean latency from TPT initiation to malignant transformation was 37.3 ± 31.3 months (95% CI 0–87.1), largely influenced by a late-onset MM diagnosed 84 months after treatment. Excluding this outlier, the mean latency fell to 21.7 months (95% CI 17.3–26.1). Both of our patients had intermediate-risk MGUS, whereas baseline risk level was generally not available in the other reports. Although publication bias may favor the reporting of plausible associations, the total number of cases remains very limited. Overall, the scarcity of signal is relatively reassuring regarding the hematologic safety of TPT in patients with MGUS.

**Table 4:** Overview of the reported cases of hematologic malignancies associated with teriparatide use

Author/year	Sex/age	Comorbidities	MGUS characteristics	Osteoporosis characteristics	Teriparatide duration	Context of malignancy detection	Hematologic malignancy (type)	Time from TPT initiation	Remarks
Forslund et al. 2007 [27]	F 57 y	Thrombophilia, occipital stroke (partial blindness), hysterectomy	Not reported	Severe OP (lumbar *T*-score −3.1), multiple VFS (L1–L4), bisphosphonate intolerance	18 months	Acute renal failure, severe hypercalcemia, massive proteinuria	MM (kappa light chain), 80% marrow infiltration	22 months	Zoledronic acid after TPT; rapid progression
Koski et al. 2009 [30]	F 59 y	None notable	Light chain kappa MGUS (590 mg/L, kappa/λ ratio 111, < 5% plasma cells) **Intermediate risk**	Severe OP (lumbar *T*-score −2.3), multiple VFs (T6–L2), vit D/calcium supplementation	18 months	Hip fracture with hypercalcemia, marrow 80% plasma cells	MM (kappa light chain)	24 months	Osteoporosis worsened (lumbar *T*-score −4.2)
Mumford et al. 2015 [39]	F 74 y	Early hysterectomy, high alcohol intake	Not reported	Severe OP (lumbar *T*-score −3.4), multiple VFs, prior bisphosphonate	2 months	Persistent severe bone pain, hypercalcemia, anemia, marrow 32% plasma cells	MM (IgA kappa)	2 months	Initially misattributed to osteoporosis; hypercalcemia before TPT
Erdemir et al. 2022 [40]	F 59 y	None notable	Not reported	Postmenopausal OP, multiple VF, prior alendronate, calcium + vit D	6 months	Back pain, hypercalcemia, proteinuria, marrow biopsy	MM (kappa light chain, marrow infiltration)	6 months	Hypercalcemia before TPT withdrawal; MM diagnosis followed
Current article Cassez et al. 2025	F/78	Systemic sarcoidosis	IgG λ MGUS, baseline M-spike 17.5 g/L **Intermediate risk**	Severe OP with multiple VFs; BMD unavailable	18 months	Bence Jones proteinuria (2014), rising M-spike, focal L1 lesion, marrow 35% plasma cells	MM (IgG λ)	84 months (7 years)	Very long delay
Current article Cassez et al. 2025	F/78	Obesity, HBP, SLE (corticosteroids)	IgM MGUS, M-spike not quantifiable, kappa/λ normal **Intermediate risk**	Severe OP with two VFs + femoral neck fracture, prolonged steroid use	24 months	Anemia, rising M-spike (5.7 g/L), marrow 20% lymphoplasmacytic cells	Waldenström macroglobulinemia (IgM)	21 months	
French PVDB case	F 71 y	TIA, type 2 diabetes	IgA kappa MGUS, monoclonal spike 6.3 g/L, urinary kappa chains **Intermediate risk**	Not reported	18 months	Anemia < 10 g/dL, CT vertebral lesions, marrow 17% plasma cells	MM (IgA kappa)	20 months	Watchful waiting adopted
French PVDB case	F 70 y	Giant cell arteritis, RAEB	Pre-existing RAEB (no MGUS)	GIOP	2 months	Worsening cytopenia, AML progression	AML	2 months	Progression of AREB to AML temporally linked to TPT

Abbreviations: *AML*, acute myeloid leukemia; *BMD*, bone mineral density; *CCI*, Charlson Comorbidity Index; *FLC*, free light chain; *GIOP*, glucocorticoid-induced osteoporosis; *HBP*, high blood pressure; *Ig*, immunoglobulin (IgA, IgG, IgM); *kappa/λ*, kappa/lambda light chains; *MGUS*, monoclonal gammopathy of undetermined significance; *MM*, multiple myeloma; *M-spike*, monoclonal protein spike (serum protein electrophoresis); *OP*, osteoporosis; *PVDB*, French pharmacovigilance database; *RAEB*, refractory anemia with excess blasts; *SLE*, systemic lupus erythematosus; *TIA*, transient ischemic attack; *TPT*, teriparatide; *VF*, vertebral fractures; *WM*, Waldenström macroglobulinemia

Among the 5 patients in whom TPT was not introduced because of MGUS, age ranged from 64 to 89 years, with highly comorbid profiles; three had IgG-type MGUS, including two classified as low risk. Despite fulfilling criteria for severe osteoporosis with multiple VF and, in some cases, very low BMD (*T*-score down to −4.8), MGUS detection systematically led to TPT exclusion and redirection to antiresorptive. This could reflect evidence that geriatric patients at very high fracture risk are often undertreated with TPT. In the German study by Rippl et al. (272,152 patients, mean age 82 years), 54% met criteria for very high fracture risk, yet only 4% received an anabolic agent, despite fewer than one-quarter having contraindications [43]. Current guidelines emphasize prioritizing osteoanabolic therapy in such patients, suggesting that some excluded in our series, notably those with low-risk IgG MGUS and very low BMD, might reasonably have benefited from TPT.

*Regarding MGUS as a risk factor for fragility-related osteopathy*, Kristinsson et al. reported in a Swedish cohort of 5326 MGUS patients a significantly increased fracture risk, particularly for axial sites, with a 10-year hazard ratio of 2.4 for vertebral and pelvic fractures [44]. This risk was independent of baseline monoclonal peak, suggesting intrinsic bone involvement. In our series, no association was found between fracture occurrence and MGUS severity, although interpretation is limited by small numbers. In our cohort, lumbar BMD increased by 6.7% (+ 0.66 SD), slightly below pivotal trials that showed 9–13% gains after 19 months of treatment [8, 10]. No significant changes were observed at the hip consistent with TPT’s predominant action on trabecular bone. Despite BMD improvement, 13.8% of patients experienced new fractures, underlining the need for larger studies with longer follow-up to better clarify fracture outcomes in this population.

*The potential skeletal benefit of TPT in MGUS-associated osteoporosis* can be supported by several elements. First, MGUS is associated with increased skeletal fragility, including up to a 2.5-fold higher risk of VF [15]. TPT has demonstrated robust antifracture efficacy in severe osteoporosis, with pivotal trials showing approximately a 65% reduction in VF together with significant improvements in lumbar spine BMD [45]. Mechanistically, MGUS-related bone disease involves inhibition of osteoblast differentiation through Wnt pathway inhibitors such as DKK1 and sclerostin [46, 47]. By reducing sclerostin levels and stimulating osteoblast activity, TPT promotes bone formation and may partially counteract this osteoblast suppression [47, 48].

**Fig. 2:**
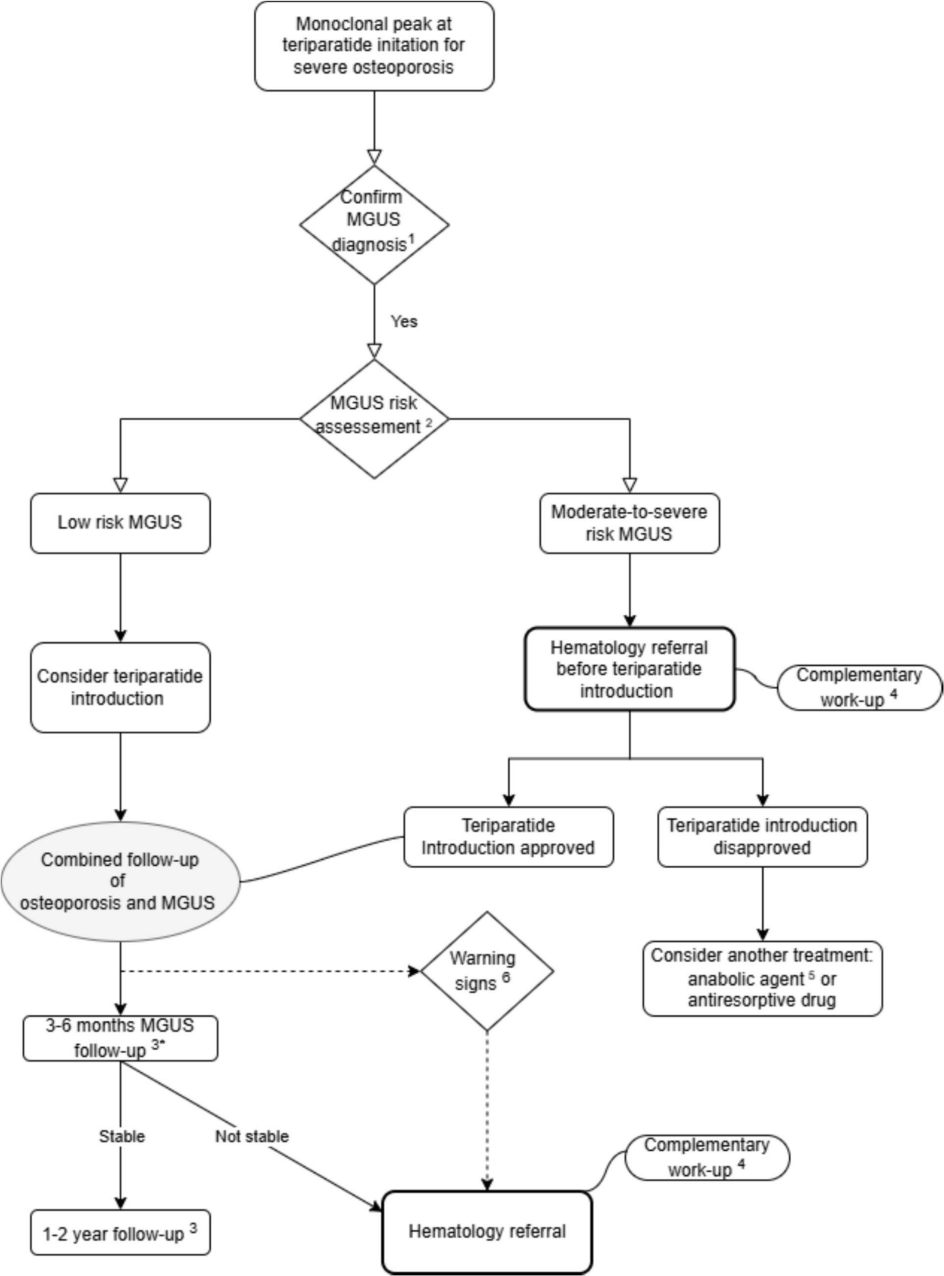
Proposition of MGUS management at teriparatide introduction. Abbreviations: MGUS = monoclonal gammapathy of undetermined significance. ^1^ International Myeloma Working Group (IMWG) criteria (IMWG 2003, Kyle R et al. 2010). ^2^Mayo Clinic classification criteria (Rajkumar et al. 2005). ^3^Biologic work-up: Complete blood count, serum creatinine, estimated glomerular filtration rate, serum albumin, corrected calcium, Urinalysis ± protein/creatinine ratio, serum immunoglobulin quantification (IgG/IgA/IgM), serum protein electrophoresis (SPE), free light chain ratio (kappa/lambda). ^4^ Complementary work-up: biology = lactate dehydrogenase (LDH), beta-2 microglobulin, N-terminal pro b-type natriuretic peptide (NT-proBNP), urinary albumin-to-creatinine ratio (RAC). Imaging = in the case of an IgM peak, a cervico-pelvic CT scan; in the case of a non-IgM peak, either a low-dose whole-body CT scan, a whole-body MRI, or a PET scan. A bone marrow biopsy is also performed. ^5^Abaloparatide, romosozumab if no contra-indication and drug available (depending on country). If not, consider antiresorptive therapy. ^6^Warning signs: anemia, kidney failure, bone pain, hypercalcemia, recurrent bacterial infections. *6-month follow-up preferred in case of low-risk MGUS, 3-month in case of medium-risk MGUS

Therefore, after considering these aspects of the benefit–risk balance, the systematic exclusion of patients with MGUS may be overly restrictive. Collaboration between hematologists and osteoporosis specialists is recommended when evaluating TPT use in this setting. The development of a consensual strategy would help standardize management and avoid systematic contraindication of TPT. A proposed clinical decision-making pathway for patients with a monoclonal peak at the time of TPT initiation is presented in Fig. 2. Following confirmation of MGUS, systematic risk assessment is advised. Low-risk patients may proceed with TPT under combined osteoporosis and MGUS follow-up (at 6 months, then every 1–2 years). Patients at intermediate or high risk should undergo hematology referral and additional work-up before approval. If TPT is initiated, closer follow-up is warranted (3 months, then every 1–2 years if stable). Any warning signs or hematologic progression require prompt hematology reassessment. Alternative osteoporosis treatments should be considered if TPT is not approved.

This study has several limitations, primarily related to its retrospective and observational design. The most significant challenge was missing data, particularly regarding BMD and MGUS risk stratification. Although the study was bicentric in design, nearly all patients (28 out of 29) were recruited from Lille University Hospital, potentially introducing a center effect. This imbalance reflects clinical practice, as very few French clinicians initiate TPT in patients with MGUS, and only in the absence of alternative therapeutic options. It is likely that MGUS patients with severe osteoporosis were managed in Angers without TPT, but their number and outcomes remain unknown, representing a major limitation of this analysis as their evaluation would have helped better address patients’ profile. The small sample size limits the statistical power and generalizability of the findings. Mostly, the absence of a control group precluded direct comparison of malignant transformation rates between TPT-exposed and unexposed patients within a MGUS cohort. Heterogeneity in treatment duration and follow-up intervals further constrained interpretation. Finally, given the rarity of hematologic progression and the study design, no causal relationship between TPT and malignant transformation can be established. Nonetheless, to the best of our knowledge, this is the largest real-world cohort evaluating MGUS patients treated with TPT. We completed this work with data from the French PVDB. Finally, by examining both osteoporosis and MGUS outcomes, this study provides clinically relevant insights for both hematologists and osteoporosis specialists.

### Conclusion

These findings suggest that progression events remain rare and might be related to the natural course of MGUS rather than to TPT exposure. TPT was effective, generally well tolerated, and associated with significant BMD gains in this multimorbid population. While caution remains warranted, especially in patients with intermediate- or high-risk MGUS, systematic exclusion may be unnecessarily restrictive. Coordinated management between osteoporosis specialists and hematologists, with appropriate risk stratification and monitoring, may allow selected patients with severe osteoporosis to benefit from anabolic therapy. Beyond TPT, newer osteoanabolic agents such as romosozumab and abaloparatide may represent alternative therapeutic options in this setting, whose safety and efficacy warrant evaluation in future studies.

## Supplementary Information

Below is the link to the electronic supplementary material.

ESM 1 (DOCX 19.3 KB)

## Data Availability

All data generated or analyzed during this study are included in this published article and its supplementary information files.
