# Supplementary material for: Malignant transformation in patients with monoclonal gammopathy of undetermined significance treated with teriparatide for osteoporosis: a bicenter retrospective study and analysis of the French national pharmacovigilance database
Source: Arch Osteoporos. 2026 Mar 29;21(1):59. doi: 10.1007/s11657-026-01693-x (PMC13033462; doi:10.1007/s11657-026-01693-x)
Supplement: Supplementary file 1 — (DOCX 19.3 KB) [file 11657_2026_1693_MOESM1_ESM.docx]

**Supplementary material**

**Supplementary Figure 1 - Therapeutic sequence after teriparatide treatment (number of patients)**

**Supplementary Table 1** – Overview of the profiles of the patients with an indication of TPT finally recused due to MGUS

| **Patient** | **Sex** | **Age at follow-up** | **Comorbidities** | **Osteoporotic fractures** | **BMD (T-score, site)** | **MGUS characteristics** | **TPT decision & alternative therapy** | **Hematologic follow-up** |
| --- | --- | --- | --- | --- | --- | --- | --- | --- |
| **1** | F | 83 | HBP, depression, polymyalgia rheumatica | Multiple VFs (L4, L5, T7, T9), FF | FN −2.1; Spine −1.8; Hip −1.8 | IgA lambda, M-spike 3.7 g/L, no Bence-Jones  **Intermediate risk (at least)** | TPT contraindicated → Risedronate switched to zoledronate | None (GP only) |
| **2** | M | 76 | Epilepsy, COPD/OSA, cognitive disorders | Multiple VFs, wrist fracture | Spine −4.8; FN −1.9; Hip −1.3 | Ig subtype not specified, M-spike 6.4 g/L  **Undetermined** | TPT contraindicated → Anti-resorptive (unspecified) | Initial hematologic, then GP |
| **3** | F | 89 | HBP, asthma, dyslipidemia | Multiple VFs (T7–T8) | Spine −0.4; Wrist −1.7 | IgM kappa, M-spike 1.4 g/L, hypogammaglobulinemia  **Intermediate risk (at least)** | TPT contraindicated → Zoledronate | None |
| **4** | F | 69 | RA, multiple peripheral fractures | Multiple VFs (T6–T7), sacrum, metatarsals | Spine −0.2; Hip −1.0 | IgG kappa, M-spike 1.5 g/L  **Low-risk** | TPT contraindicated → Denosumab | Not documented |
| **5** | F | 64 | Thyroid disease, menopause at 50, former smoker | Multiple VFs (L4, later L5) | Spine −4.1; FN −2.1; Hip −2.4 (2019); Spine −3.3 (2023) | IgG kappa, M-spike 3.6 g/L, normal FLC ratio  **Low-risk** | TPT contraindicated → Alendronate → Zoledronate, switch planned to Denosumab | None |

Abbreviations: BMD = bone mineral density; COPD = chronic obstructive pulmonary disease; FLC = free light chain; FN = femoral neck; GP = general practitioner; HBP = high blood pressure; Ig = immunoglobulin (IgA, IgG, IgM); MGUS = monoclonal gammopathy of undetermined significance; M-spike = monoclonal protein spike (serum protein electrophoresis); OP = osteoporosis; OSA = obstructive sleep apnea; RA = rheumatoid arthritis; TPT = teriparatide; VF = vertebral fractures.
